# Mechanisms and molecular characterization of relapsed/refractory neuroblastomas

**DOI:** 10.3389/fonc.2025.1555419

**Published:** 2025-03-06

**Authors:** Chong Chen, Zixuan Wei

**Affiliations:** ^1^ Department of Clinical Laboratory, Tianjin Union Medical Center, The First Affiliated Hospital of Nankai University, Tianjin, China; ^2^ Department of Pediatric Oncology, Tianjin Medical University Cancer Institute and Hospital, National Clinical Research Center for Cancer, Tianjin, China; ^3^ Key Laboratory of Cancer Prevention and Therapy, Tianjin, China; ^4^ Department of Pediatric Oncology, Tianjin’s Clinical Research Center for Cancer, Tianjin, China; ^5^ Department of Pediatric Oncology, Key Laboratory of Cancer Immunology and Biotherapy, Tianjin, China

**Keywords:** neuroblastoma, relapsed/refractory, molecular characterization, mechanism, targeted therapy, precision oncology

## Abstract

Relapsed/refractory neuroblastoma is a type of malignant solid tumor with a very poor prognosis in children. Its pathogenesis is complex, involving multiple molecular pathways and genetic alterations. Recent studies have shown that MYCN amplification, ALK mutation, TERT promoter mutation, p53 pathway inactivation, and chromosomal instability are the key mechanisms and molecular characteristics of relapsed/refractory neuroblastoma. Precision treatment strategies targeting these molecular mechanisms have shown certain prospects in preclinical studies and clinical practice. This review focuses on the relevant mechanisms and molecular characteristics of relapsed/refractory neuroblastoma, explores its relationship with treatment response and clinical prognosis, and briefly introduces the current treatment strategies to provide a theoretical basis for the development of novel and personalized therapeutic regimens to improve the prognosis of children.

## 1 Introduction

Primary neuroblastoma originates from neural crest progenitor cells, cells of the sympathetic nervous system in the adrenal medulla or sympathetic chain (1–3). Neuroblastomas are remarkably heterogeneous, with unique and variable biological and clinical features, including limited disease that can spontaneously regress and widespread disseminated disease (4, 5). Based on this heterogeneity, neuroblastomas are stratified into low to intermediate risk tumors that can be surgically resected and high-risk tumors that are fatal. About half of diagnosed neuroblastomas are classified as high-risk cases (6).

Although the efficacy of multimodal therapies has been gradually improving in recent years, the 5-year survival rate for patients with high-risk neuroblastoma has only increased to approximately 50% (7). Within this group, there is also a subset of patients with a particularly poor prognosis who do not respond to initial treatment or who do not achieve complete remission, accounting for about 10-20% of children with high-risk neuroblastoma, called refractory neuroblastoma. In addition, about 40-50% of children with neuroblastoma whose disease goes into remission after treatment will eventually have the cancer relapse during or after treatment, called relapsed neuroblastoma (8–10). These tumors are usually aggressive and resistant to conventional therapies such as chemotherapy or radiation. The 5-year overall survival rate for patients with relapsed/refractory neuroblastoma is less than 20% (11). Clinically, the treatment options for these two types of tumors are usually the same, so they are generally referred to collectively as relapsed/refractory neuroblastomas.

**Figure 1:**
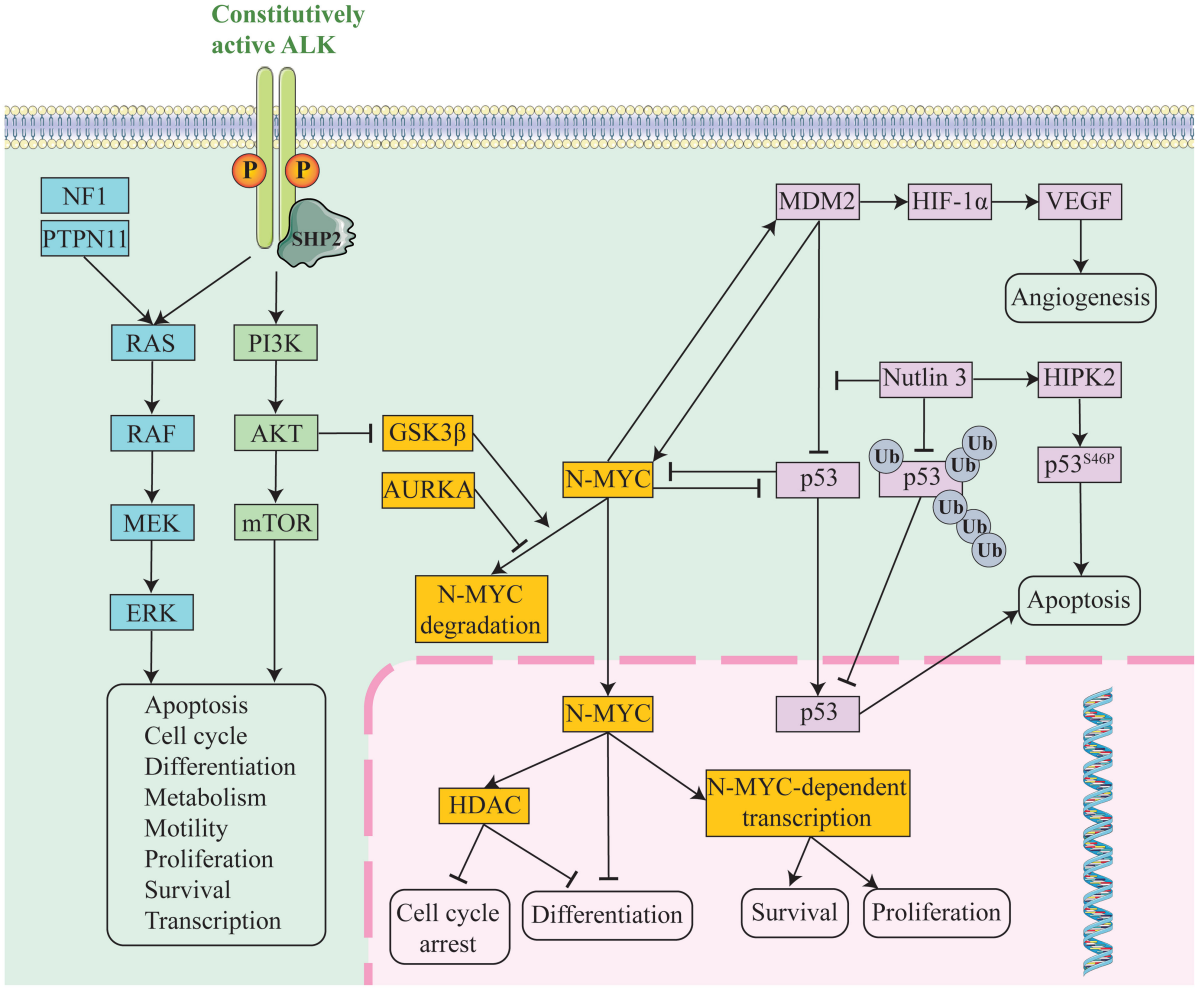
Conceptual diagram of molecular pathway mechanisms in relapsed/refractory neuroblastoma. RAS pathway in blue, PI3K pathway in green, MYCN pathway in orange and p53 pathway in pink.

**Figure 2:**
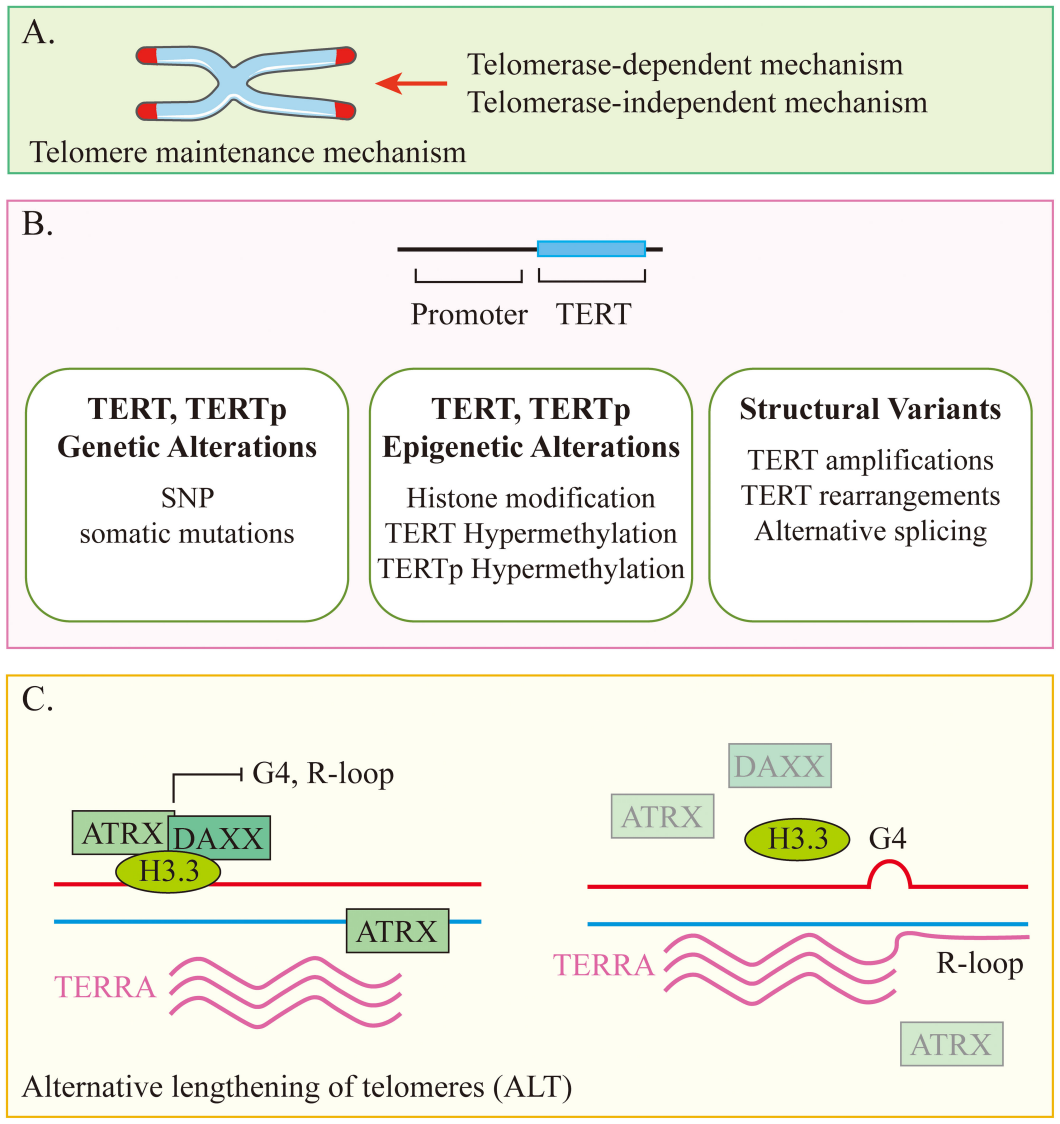
Conceptual diagram of the telomere maintenance mechanism. **(A)** Telomere maintenance mechanisms can be divided into two types based on whether or not they rely on telomerase. **(B)** Telomerase-dependent mechanism. **(C)** Telomerase-independent mechanism.

The development of neuroblastoma reflects an abnormal developmental process of adrenal sympathetic nerve cells. Somatic variants are uncommon in neuroblastoma, whereas copy number variants across segmental chromosomal regions or even entire chromosomes are common (12). Molecular analytical studies have revealed key factors in the biology and treatment resistance of aggressive tumors, while providing important guidance for the development of new therapies. In this review, we discussed the pathogenesis of relapsed/refractory neuroblastoma and summarized some of the molecular characteristics that may be used to predict prognosis or as therapeutic targets (**Table 1**). In addition, we have summarized the current status of the application of drugs targeting these therapeutic targets (**Table 2**). In order to understand the molecular mechanisms of relapsed/refractory neuroblastoma more intuitively, we have drawn corresponding conceptual diagrams for better reading (**Figures 1**, **2**).

**Table 1:** Molecular characterization and targeted drugs in relapsed/refractory neuroblastoma.

Molecular characterization	Candidate gene	Targeted drugs
ALK mutations or gene amplification	ALK	ALK tyrosine kinase inhibitors: Crizotinib, Ceritinib, Lorlatinib
MYCN amplification	MYCN	BET inhibitor: GSK525762 (I-BET726) HDAC inhibitor: Vorinostat Dual HDAC/PI3K inhibitor: CUDC-907 Aurora A kinase inhibitor: Alisertib (MLN8237), SK2188
Activation of telomere maintenance mechanisms	TERT, ATRX, MYCN	Telomerase inhibitor: Imetelstat (GRN163L), BIBR-1532, sodium metaarsenite (KML001), Telomestatin, 6-thio-2′-deoxyguanosine (6-thio-dG), XAV939 ATM inhibitor: AZD0156 ATR inhibitor: AZD6738
p53 signalling pathway alterations	p53, MDM2, p21, p14	MDM2 antagonist: RG7388 (Idasanutlin), MI-773 (SAR405838) MDM2 inhibitor: HDM-201, MI-63, RITA, SP141 Dual MDM2/MDMX inhibitor: ALRN-6924 MDM2/p53 interaction inhibitor: Nutlin-3
RAS signalling pathway alterations	RAF, MEK, ERK, PI3K, AKT, NF1, PTPN11, ALK, BRAF, FGFR1, SHP2	MEK inhibitor: Binimetinib, Trametinib SHP2 inhibitor: SHP099, II-B08, RMC-4550
Deletion of chromosome 1p	KIF1Bb, CHD5, miR-34a, ARID1A, CAMTA1	
Deletion of chromosome 6q21	SFT2D1, UNC93A, MLLT4	
Deletion of chromosome 11q	DLG2, CADM1, H2AFX, ATM, CHK1, MRE11, CCND1	

**Table 2:** The application of targeted agents for relapsed/refractory neuroblastoma.

Target	Drug	*In vitro* and *in vivo* activities	Clinical trial	Ref
ALK	Crizotinib	• The objective response rate in patients with relapsed/refractory neuroblastoma was 15%. • The objective response rate in pediatric patients with solid tumors was 17%.	Phase 1, 2. (NCT00939770); Phase 1. (NCT01606878)	(1)
	Ceritinib	• Neuroblastoma cell lines are sensitive to this inhibitor. • The inhibitor was able to significantly induce apoptotic effects in an *in vivo* neuroblastoma mouse model. • This inhibitor in combination with CDK4/6 inhibitors effectively enhances growth inhibition, promotes cell cycle arrest and induces cell death. • Among patients with ALK-positive neuroblastoma, 20% achieved an overall response.	Phase 1. (NCT02780128); Phase 1. (NCT01742286)	(2, 3)
	Lorlatinib	• Lorlatinib showed excellent preclinical activity in ALK-driven models independent of ALK gene mutation hotspots. • In the phase 1 trial, patients achieved robust and sustained responses regardless of underlying ALK gene mutations. The response rate in patients younger than 18 years of age was 30% for single agent and 63% for combination chemotherapy.	Phase 1. (NCT03107988); Phase 3. (NCT03126916)	(4)
BET	GSK525762 (I-BET726)	• BET inhibitors promote apoptosis and directly inhibit BCL2 and MYCN in an *in vitro* cell line. Oral administration of inhibitors inhibits tumor growth in a mouse xenograft model. • The combined inhibition of BET and MEK demonstrated synergistic effects in the majority of neuroblastoma cell lines under *in vitro* conditions, but exhibited limited antitumor activity *in vivo*.		(5, 6)
HDAC	Vorinostat	• The response rate for patients receiving the combination of Vorinostat and MIBG was 32%. • Combination therapy with anti-GD2 antibody and Vorinostat significantly inhibits tumor growth in an aggressive *in situ* model of neuroblastoma.	Phase 1. (NCT00217412); Phase 1. (NCT01019850)	(7–12)
HDAC/PI3K	CUDC-907	• The inhibitor significantly inhibited the proliferation and colony formation and was able to induce apoptosis and block the cell cycle in neuroblastoma cell lines.		(13, 14)
Aurora A kinase	Alisertib (MLN8237)	• In patients with relapsed/refractory neuroblastoma, the overall response rate to Alisertib treatment in combination with irinotecan and temozolomide was 31.8%. • In children with relapsed/refractory neuroblastoma or acute leukemia, the objective response rate to Alisertib single-agent therapy was less than 5%.	Phase 1,2. (NCT01601535); Phase 2. (NCT01154816)	(15–17)
Telomerase	Imetelstat (GRN163L)	• Treatment of xenograft tumors with imetelstat as a single agent resulted in a reduction of telomerase activity by approximately 50% and a significant increase in survival compared to controls. • The combination of imetelstat and etoposide provides a synergistic effect. • The most common toxicities in phase 1 trials were neutropenia, thrombocytopenia, and lymphopenia.		(18, 19)
	6-thio-2′-deoxyguanosine (6-thio-dG)	• Significant synergistic anti-tumor effects were noted when 6-thio-dG was combined with either etoposide or doxorubicin in telomerase-positive neuroblastoma cell lines during *in vitro* studies.		(18)
ATM	AZD0156	• AZD0156 reverses resistance to temozolomide + irinotecan in ALT neuroblastoma models.		(20)
ATR	AZD6738	• ALT neuroblastoma cells exhibit greater resistance to the clinical ATR inhibitor AZD6738 when compared to other subtypes of neuroblastoma.		(21)
MDM2	RG7388 (Idasanutlin)	• Neuroblastoma cell lines, especially p53 wild-type cell lines, are sensitive to this inhibitor. • The inhibitor was able to significantly induce apoptotic effects in an *in vivo* neuroblastoma mouse model. • Combination with other inhibitors significantly inhibits tumor cell viability and enhances antitumor activity.	Phase 1,2. (NCT04029688)	(22–24)
	MI-63	• Neuroblastoma cell lines, especially MYCN-expanded cell lines, are sensitive to this inhibitor. The inhibitor has a significant inhibitory effect on the growth activity of tumor cells, induces apoptosis and mediates G1 phase block. • Combination with vincristine enhances vincristine-mediated growth inhibition when used in p53-mutant neuroblastoma cell lines.		(25, 26)
	MI-773 (SAR405838)	• Neuroblastoma cell lines, especially p53 wild-type cell lines, are sensitive to this inhibitor. • The inhibitor was able to significantly induce apoptotic effects in an *in vivo* neuroblastoma mouse model. • Enhances doxorubicin cytotoxicity in neuroblastoma cell lines.		(27, 28)
	HDM-201	• In the Phase I trial, the overall response rate at the recommended expanded dose was 10.3%.	Phase 1. (NCT02143635)	(29)
MDM2/MDMX	ALRN-6924	• ALRN-6924 was first studied in a Phase 1 trial in children and no results have been published.	Phase 1. (NCT03654716)	
MDM2/p53	Nutlin-3	• Neuroblastoma cell lines, especially MYCN-expanded cell lines, are sensitive to this inhibitor. The inhibitor has a significant inhibitory effect on the growth activity of tumor cells, induces apoptosis and mediates G1 phase block. • Induction of p53 pathway activation and apoptosis in a mouse model of p53 wild-type tumor xenografts. • Combinations with other inhibitors significantly inhibit cell viability.		(25, 30)
	Alrizomadlin (APG-115)	• Alrizomadlin had an acceptable safety profile and demonstrated promising antitumor activity in MDM2-amplified and TP53 wild-type tumors.	Phase 1. (CTR20170975)	(31)
MEK	Binimetinib	• Binimetinib is effective in preclinical models of neuroblastoma tumors with low NF1 expression. • Binimetinib and CDK4/6 inhibitors are synergistic in preclinical models of neuroblastoma.		(32, 33)
SHP2	SHP099	• SHP099 significantly inhibited tumor cell proliferation in preclinical models. • Neuroblastoma cell lines, especially those with NF1 deletion or low expression, are highly sensitive to SHP099.		(34, 35)

## 2 Recurrent somatic alterations

### 2.1 ALK

Anaplastic lymphoma kinase (ALK) is a receptor tyrosine kinase (RTK) whose activation generates mitogenic signaling through the RAS-MAPK and PI3K-AKT pathways (13). Point mutations, copy number amplifications, or chromosomal translocations within the structural domain of ALK tyrosine kinase can lead to oncogenic activation. ALK mutations or gene amplification are present in up to 15% of sporadic high-risk neuroblastomas, the most common somatic single-nucleotide variant in neuroblastoma and the most frequently mutated oncogene (**Figure 1**). The data suggest that high-risk neuroblastoma patients with ALK variants are a particularly high-risk group (14). In the SIOPEN high-risk group, ALK variants were categorized as clonal or subclonal. The difference in OS between cases with ALK amplification or clonal ALK variants and cases with subclonal ALK variants or no ALK changes was statistically significant. In multivariate models, ALK amplification and clonal ALK variants were independent predictors of poor prognosis (15). In the COG high-risk neuroblastoma population, a poorer prognosis has also been observed in cases with ALK variants or ALK amplification (14). In addition, ALK mutations can contribute to disease recurrence. Increased somatic mutations and increased ALK-activating subclonal/clonal mutations have been reported in relapsed neuroblastomas compared to tumors at diagnosis; the frequency of these mutations is higher than 20% and rises as tumors and/or plasma from relapsed patients are sequenced more routinely (10, 16, 17).

ALK tyrosine kinase structural domain variants occur mainly at three hotspot positions (positions F1174, R1275, and F1245), with 10% to 15% of the variants occurring at other kinase structural domain positions (18). The data suggest that MYCN gene amplification is often accompanied by ALK mutation activation, and the two synergistically initiate and promote the development of neuroblastoma (19, 20). At the molecular level, ALK induces MYCN transcription and stabilizes MYCN protein; conversely, MYCN increases ALK transcription (21, 22). Interestingly, a higher frequency of the F1174L mutation was observed in MYCN-amplified tumors (23). ATP-competitive ALK/Met/ROS1 tyrosine kinase inhibitors (TKIs), such as crizotinib, exhibit differential activity in preclinical models of ALK-driven neuroblastoma (24). This class of drugs primarily induces remission in patients with the ALK R1275 mutation. Primary resistance to inhibitors such as crizotinib is commonly associated with ALK hotspot mutations (e.g., F1174L and F1245C) in neuroblastomas, hence the term refractory ALK variants for such variants (14, 24). Targeting drug-resistant ALK mutations, the third-generation ALK tyrosine kinase inhibitor loratinib exerted unprecedented preclinical activity as a single agent in a xenograft model of neuroblastoma patients with three hotspot mutations (25). The first phase I study of the New Approaches to Neuroblastoma Therapy Consortium (NANT2015-02) in children (NCT03107988) explored the use of loratinib in patients with ALK-driven refractory or relapsed neuroblastoma, demonstrating the drug’s safety profile and significant clinical activity. Esther R’s team showed that the acquisition of ALK compound mutations and mutations in members of the RAS-MAPK pathway is the mechanism by which ALK-driven neuroblastoma patients develop resistance to loratinib (26). Among them, mutations in the RAS-MAPK pathway are the most common mechanism of off-target resistance to loratinib (27). Loratinib is currently enrolled in the Children’s Oncology Group (COG) (NCT03126916) and the International Society of Pediatric Oncology Europe Neuroblastoma Group (SIOPEN) phase 3 trials to further understand clinical response and drug resistance in patients (28).

### 2.2 MYCN alteration

MYCN, located on chromosome 2p24, is a major transcriptional regulator of cell growth, metabolism and differentiation. Approximately 50% of high-risk patients present with MYCN amplification, and these tumors tend to be the most aggressive and difficult to treat (29). High level of MYCN amplification is a driver of high-risk neuroblastoma. This is associated with reduced tumor immunogenicity and can promote tumor metastasis and recurrence (12). In addition, MYCN amplification inhibits interferon activity and chemokine expression, and its overexpression enhances tumor cell resistance to immune-mediated cytotoxicity through a variety of mechanisms, including MHC-I downregulation and inhibition of NK cell activation (30). In addition to functioning as a transcription factor, MYCN promotes disease progression and metastasis through epigenetic regulation (**Figure 1**) (31). In almost all multivariate regression analyses of prognostic factors, regardless of disease stage, MYCN gene amplification strongly predicted a poorer prognosis, including treatment response rate, time to tumor progression, and overall survival. Of the 4832 newly diagnosed patients enrolled in the ANBL00B1 (NCT00904241) study, patients harboring MYCN-unamplified tumors (n = 3647; 81%) had 5-year event-free survival (EFS) and overall survival (OS) rates of 77% and 87%, respectively. Patients with MYCN amplification (n = 827; 19%) had 5-year EFS and OS rates of 51% and 57%, respectively (32). In the cohort of 6,223 patients with known MYCN status in the INRG database, the hazard ratio for OS associated with MYCN amplification was 6.3. The greatest adverse prognostic impact of MYCN amplification on OS was seen in younger patients (age <18 months) (33). On this point, it has been shown that the presence of MYCN amplification appears to have a greater adverse prognostic impact in patients with other favorable characteristics (e.g., younger age and lower stage), whereas the prognostic impact of MYCN amplification is less severe in older patients with higher disease stage. However, MYCN amplification still has a negative prognostic impact even in high-risk patients, who tend to respond poorly to conventional chemotherapy and require treatment with high-dose chemotherapy and autologous stem cell transplantation (33, 34).

Therapeutic strategies targeting MYCN through its downstream targets include bromodomain and extra-terminal domain (BET) inhibition, dual HDAC/PI3K inhibition, MDM2 inhibition and aurora A kinase inhibition. Various BET inhibitors are being used in clinical trials, such as a phase I study of GSK525762 (I-BET726) in neuroblastoma. In addition, the HDAC inhibitor vorinostat has been studied in several clinical trials in patients with NB (NCT00217412, NCT01132911, NCT02035137, NCT02559778, NCT01019850 and NCT01208454) (35–40).

### 2.3 Activation of telomere maintenance mechanisms

Telomeres are regions of repetitive nucleotide sequences (TTAGGG) located at the ends of chromosomes. In normal dividing cells, telomeres gradually shorten with each cell replication until they reach a critical level, eventually causing the cell to be unable to replicate, and cellular senescence occurs (41). Conversely, lengthening telomeres promotes cell survival, which is known as telomere maintenance mechanism (TMM). Activation of TMM to prevent telomere shortening is necessary for the continued proliferation of cancer cells, and patients whose tumors possess TMM have a poor prognosis.

Telomerase is able to maintain telomere length by adding telomeric DNA repeats. It is a reverse transcriptase enzyme that consists of the catalytic protein subunit TERT and human telomerase RNA (hTR). Most cancers overexpress telomerase, which is usually associated with overexpression of TERT (42, 43). Alternative lengthening of telomeres (ALT) is the maintenance of telomeres in the absence of telomerase activity. In neuroblastoma, there is a strong association between ALT and loss of function (LoF) gene variants in ATRX (**Figure 2**) (44). Neuroblastoma cells maintain telomere length through one of several mutually exclusive mechanisms, and these tumors typically have poor response rates and poor clinical outcomes (45, 46). MYCN amplification is present in nearly 40% of high-risk neuroblastomas and is associated with upregulated expression of TERT and telomere dysfunction (47, 48). In another 23%-31% of high-risk neuroblastomas, TERT is activated through proximal chromosomal rearrangements, which induces its transcriptional upregulation (47, 49). ALT is also active in about 24% of high-risk neuroblastomas, about half of which are associated with somatic alterations in ATRX (47, 49, 50). A higher proportion of ALT-positive neuroblastoma cases were found in the relapsed patient cohort compared to the newly diagnosed cohort (10% and 48%, respectively). Patients with ALT-positive tumors also have as poor event-free survival as patients with MYCN amplification, while their long-term survival rates are very low and usually unsalvageable after progression or relapse (45, 51). In conclusion, a growing body of data supports TMM as one of the mechanisms and molecular features of most aggressive high-risk neuroblastomas that relapse or are refractory to treatment.

Drugs that target telomerase activity include imestat (GRN163L), BIBR-1532, and sodium metaarsenite (KML001), but all of these drugs suffer from excessive toxic effects. Telomestatin, 6-thio-2′-deoxyguanosine (6-thio-dG), and XAV939 have shown the ability to induce apoptosis in neuroblastoma cells in cell lines and preclinical models, but further clinical development is still needed (52). Drugs targeting ALT include ataxia-telangiectasia mutated (ATM) inhibitor combinations such as the ATM inhibitor AZD0156. Using cell lines and *in vitro* models derived from patients with relapsed neuroblastoma, the study by Balakrishna Koneru et al. found that constitutive ATM activation in ALT-positive cells contributes to the chemoresistant phenotype of neuroblastoma. In contrast, AZD0156 reversed resistance to temozolomide and irinotecan in ALT-positive neuroblastoma cell lines and xenograft models, providing a rationale for early clinical trials (53). Other ALT-related drugs are ataxia telangiectasia and Rad3-related (ATR) inhibitors such as AZD6738 (54). Although activation of the TMM has been shown to be a key factor in the poor prognosis of neuroblastoma, MYCN amplification and ATRX mutations, the primary drivers of the TMM, are also associated with multiple modes of transcriptional activation that drive malignant transformation. In addition, neuroblastoma patients with mutations in both TMM and the RAS/TP53 pathway have a particularly poor prognosis (55). As a result, the likelihood that aggressive relapsed/refractory neuroblastomas will be resistant to single agents targeting TMM is high, and therapeutic strategies targeting TMM will only show significant clinical efficacy in combination with agents targeting these key genes and pathways. For example, *in vitro* experiments targeting neuroblastoma by Janina Fischer-Mertens et al. demonstrated that 6-thio-dG and the competitive telomerase inhibitor, imestat, were more efficacious than monotherapy when combined with other agents such as etoposide (56).

### 2.4 p53 signalling pathway alterations

The p53 is a key regulator of cell cycle checkpoints and apoptosis. Activated after cellular stress, it binds DNA in a sequence-specific manner and activates the transcription of a large number of downstream genes (including MDM2, p21, etc.), leading to apoptosis, cell cycle arrest, differentiation and DNA repair (**Figure 1**) (57). Mutational inactivation of the p53 gene occurs in more than half of human malignancies. Amplification of MDM2 also occurs in neuroblastoma, and even in the absence of gene amplification, MDM2 protein overexpression is often present and correlates with a poorer prognosis for patients. MDM2, located upstream of p53, is a negative regulator of p53 and acts as a ubiquitin ligase, targeting p53 for proteasome-mediated degradation, forming an autoregulatory feedback loop that tightly regulates the cellular level of p53. MDM2 also inhibits the activity of p53 by increasing the degradation of p53, contributing to tumor formation (58). MDM2 also functions independently of p53 to promote the growth, progression and development of neuroblastoma (59). For example, elevated MDM2 expression promotes multidrug resistance in neuroblastoma, leading to relapsed/refractory neuroblastoma (60). Mutations in the p53 signaling pathway occur in less than 2% of patients with a primary diagnosis of neuroblastoma (61). In contrast, in relapsed neuroblastoma after chemotherapy, the frequency of mutations in the p53/MDM2/p14ARF signaling pathway increases to almost half and leads to chemotherapy resistance (62, 63). The association of p53 mutations in neuroblastoma cells with drug resistance has been demonstrated, and these cell lines are more chemoresistant than p53 wild-type neuroblastoma cell lines (61).

Since most neuroblastomas harbor functional wild-type p53, it may be wise to target MDM2 to enhance the functional activity of p53. The MDM2 antagonist idasanutrin has shown strong antitumor effects in preclinical models (64). In addition, another MDM2 antagonist in clinical trials, MI-773 (SAR405838), has been shown to potentiate the cytotoxicity of doxorubicin in neuroblastoma cell lines (65). There are also antagonists targeting the interaction of MDM2 and p53, such as Nutlin-3, which activate the p53 pathway in chemoresistant neuroblastoma with wild-type p53, inhibit primary tumor growth, and reduce tumor metastasis in mice carrying chemoresistant neuroblastoma xenografts (66). An increasing number of targeted agents have entered early pediatric trials, such as HDM-201 (NCT02780128), an MDM2 inhibitor applied to neuroblastoma, and ALRN-6924 (NCT03654716), a dual MDM2/MDMX inhibitor applied to solid tumors in children.

### 2.5 RAS signalling pathway alterations

Members of the RAS protein family are GDP-GTP-regulated switches that regulate the cytoplasmic-nuclear signaling network that controls normal cellular processes. They send signals through a series of molecular pathways (such as RAF/MEK/ERK and PI3K/AKT, etc.) to regulate cell survival, proliferation, and differentiation (**Figure 1**). With constitutively activating mutations in the RAS family of genes in up to 30% of cancers, dysregulation of RAS-dependent signaling is essential for tumorigenesis (67). Mutations in the RAS pathway occur frequently in neuroblastoma, not only in the RAS gene itself, but also through mutations in regulatory proteins and downstream signaling components (e.g., NF1 and PTPN11) or by triggering constitutive activation of the receptor kinases of the pathway (e.g., ALK) (68). RAS pathway mutations in neuroblastoma, especially in high-risk patients, are strongly associated with poor prognosis. Patients harboring RAS pathway mutations have a worse prognosis than those harboring ALK mutations. Patients with both RAS pathway mutations and telomere maintenance mechanisms have an extremely poor prognosis (55). It was found that the frequency of mutations in the RAS-MAPK signaling pathway was significantly increased in relapsed neuroblastoma tumors, including mutations in ALK, NF1, BRAF, PTPN11, FGFR1, and the three RAS genes, and that the mutations in this pathway were mutually exclusive (69).

Targeting the RAS signaling pathway has been a challenge, but effective therapies applied to inhibit RAS-driven neuroblastoma have not been developed for more than three decades (70). Therefore, attempts to target the RAS pathway have focused on inhibiting its upstream or downstream effectors.MEK is an effector molecule of RAF, and *in vitro* experiments have found that neuroblastoma cell lines with mutations in the RAS family are sensitive to MEK inhibitors, but the presence of MYCN amplification leads to resistance to this class of drugs. The tyrosine phosphatase SHP2 encoded by PTPN11 is an activator of RAS. SHP2 inhibitors alone are susceptible to resistance in neuroblastoma, however, dual inhibition of SHP2 and the RAS effectors RAF, MEK, or ERK demonstrated synergistic effects (71). Therefore, combinations of drugs targeting this pathway may be an effective strategy for the treatment of relapsed/refractory neuroblastoma. A single-agent clinical trial of trametinib as a treatment for patients with RAS-mutated relapsed neuroblastoma is currently underway (NCT02780128).

## 3 Chromosomal alterations

Chromosomal alterations are very common in neuroblastoma, occurring in about 90% of patients, and there is a clear correlation between the type of alteration and prognosis (72). In particular, large segmental chromosome imbalances and localized aberrations are common in high-risk tumors, and any type of segmental chromosome aberrations is associated with a poorer prognosis, perhaps because loss of segmental chromosomes leads to inactivation of tumor suppressor genes. Relapsed neuroblastoma often occurs with deletions of chromosome 1p or 6q21 (73). Tumor suppressor genes located at 1p36 may include KIF1Bb, CHD5, miR-34a, ARID1A and CAMTA1 (74, 75). Among them, it has been shown that deletion of ARID1A promotes cell invasion and migration and causes neuroblastoma cells to exhibit enriched mesenchymal-type gene features (73). Pauline Depuydt et al. showed that neuroblastomas in the presence of a deletion in the distal region of chromosome 6q are highly aggressive and highly susceptible to developing into relapsed/refractory tumors (76). They also identified a number of candidate genes located in this region that were strongly associated with prognosis, such as SFT2D1, UNC93A, and MLLT4. In children younger than 18 months, only segmental chromosome aberrations led to relapse and death, and 11q deletion was the strongest prognostic marker (77). In addition, patients with heterozygous deletions of 11q are less likely to respond to induction therapy, thus promoting the development of refractory neuroblastoma (78). Many studies have been conducted on genes within the deletion region of chromosome 11q, including DLG2, CADM1, H2AFX, ATM, CHK1, MRE11, and CCND1, which are tumor suppressor genes. Many of these belong to cell growth control regulatory genes or DNA repair genes, which favor tumor development in the presence of haploinsufficiency (79–81).

## 4 Cell state

The state of neuroblastoma cells significantly influences treatment response and prognosis. Based on RNA sequencing and epigenomic analyses, a method has been proposed to describe cell states, classifying neuroblastoma cells into a differentiated adrenergic cell population and a less differentiated mesenchymal cell population (82–86). These two cancer cell states have different gene expression profiles and are dynamic and programmable to transform into each other (73, 87, 88). For example, deletion of chromosome 1p, leading to loss of the tumor suppressor gene ARID1A, promotes the development of the mesenchymal state (73). Adrenergic cells are generally more sensitive to initial treatments, whereas mesenchymal cells exhibit stronger resistance to cytotoxic chemotherapy (82). Enrichment of mesenchymal gene expression signatures has been observed in relapsed samples. These data suggest that cellular states influence treatment sensitivity (82, 86, 87, 89, 90). Under therapeutic pressures such as chemotherapy or radiation, adrenergic cells may transition to the mesenchymal state, enabling tumor cells to acquire enhanced survival capabilities and drug resistance (91). This phenotypic plasticity plays a critical role in the development of relapsed/refractory neuroblastoma.

## 5 Conclusion

Relapsed/refractory neuroblastoma occurs by complex mechanisms involving multiple genetic, epigenetic, and tumor microenvironmental changes. Currently, despite our initial understanding of their molecular characterization, there is a lack of effective therapeutic tools to deal with their relapse and drug resistance. These tumors often exhibit genetic mutations, chromosomal instability, and aberrant activation of key pathways, leading to poor therapeutic response and poor clinical prognosis. In-depth study of these molecular mechanisms can help reveal new therapeutic targets and provide patients with more precise and personalized treatment options. Future research should focus on the discovery of new biomarkers, improvement of existing therapeutic strategies, and exploration of the clinical application of innovative therapeutic tools such as immunotherapy and targeted therapy, in the hope of bringing new breakthroughs in the treatment of relapsed/refractory neuroblastoma.

Our understanding of relapsed/refractory neuroblastoma will continue to deepen as molecular biology and genomics technologies continue to evolve. Future studies will rely more on advanced methods such as high-throughput sequencing technology, single-cell genomics, and tumor microenvironmental analysis to fully resolve the interactions between tumor cells and the surrounding environment. In addition, the development of gene editing technologies and immunotherapy offers new possibilities for neuroblastoma treatment, especially in targeting tumor-associated gene mutations, immune escape mechanisms, and cellular drug resistance interventions. Through the combined application of these innovative strategies, we expect to achieve more effective personalized treatment in the future and improve the survival and quality of life of patients with relapsed/refractory neuroblastoma.
